# Association between high-density lipoprotein cholesterol and type 2 diabetes mellitus: dual evidence from NHANES database and Mendelian randomization analysis

**DOI:** 10.3389/fendo.2024.1272314

**Published:** 2024-02-22

**Authors:** Zhaoqi Yan, Yifeng Xu, Keke Li, Liangji Liu

**Affiliations:** ^1^ Jiangxi University of Traditional Chinese Medicine, Graduate School, Nanchang, Jiangxi, China; ^2^ Affiliated Hospital of Jiangxi University of Traditional Chinese Medicine, Department of Respiratory and Critical Care Medicine, Nanchang, Jiangxi, China

**Keywords:** high-density lipoprotein cholesterol, type 2 diabetes mellitus, the national health and nutrition examination survey, Mendelian randomization, causal relationship

## Abstract

**Background:**

Low levels of high-density lipoprotein cholesterol (HDL-C) are commonly seen in patients with type 2 diabetes mellitus (T2DM). However, it is unclear whether there is an independent or causal link between HDL-C levels and T2DM. This study aims to address this gap by using the The National Health and Nutrition Examination Survey (NHANES) database and Mendelian randomization (MR) analysis.

**Materials and methods:**

Data from the NHANES survey (2007-2018) with 9,420 participants were analyzed using specialized software. Logistic regression models and restricted cubic splines (RCS) were used to assess the relationship between HDL-C and T2DM incidence, while considering covariates. Genetic variants associated with HDL-C and T2DM were obtained from genome-wide association studies (GWAS), and Mendelian randomization (MR) was used to evaluate the causal relationship between HDL-C and T2DM. Various tests were conducted to assess pleiotropy and outliers.

**Results:**

In the NHANES study, all groups, except the lowest quartile (Q1: 0.28-1.09 mmol/L], showed a significant association between HDL-C levels and reduced T2DM risk (all P < 0.001). After adjusting for covariates, the Q2 [odds ratio (OR) = 0.67, 95% confidence interval (CI): (0.57, 0.79)], Q3 [OR = 0.51, 95% CI: (0.40, 0.65)], and Q4 [OR = 0.29, 95% CI: (0.23, 0.36)] groups exhibited average reductions in T2DM risk of 23%, 49%, and 71%, respectively. In the sensitivity analysis incorporating other lipid levels, the Q4 group still demonstrates a 57% reduction in the risk of T2DM. The impact of HDL-C levels on T2DM varied with age (P for interaction = 0.006). RCS analysis showed a nonlinear decreasing trend in T2DM risk with increasing HDL-C levels (P = 0.003). In the MR analysis, HDL-C levels were also associated with reduced T2DM risk (OR = 0.69, 95% CI = 0.52-0.82; P = 1.41 × 10^-13^), and there was no evidence of pleiotropy or outliers.

**Conclusion:**

This study provides evidence supporting a causal relationship between higher HDL-C levels and reduced T2DM risk. Further research is needed to explore interventions targeting HDL-C levels for reducing T2DM risk.

## Introduction

Diabetes mellitus (DM) is currently at an unimaginable level of prevalence, with an estimated 439 million adult diabetes patients by 2030 (1), of which over 90% are Type 2 diabetes mellitus (T2DM) patients. The risk factors for T2DM stem from complex genetic (polygenic) and environmental factors, making the disease familial and racially clustered. It is also associated with age, obesity, diet, and lack of physical activity (2). Despite the recognition of T2DM as the main culprit for various cardiovascular and cerebrovascular diseases, complications often arise too late, and there is currently no cure for the disease. Therefore, it is of great significance to work on prevention and delaying the progression of T2DM. In addition to blood glucose changes, the clinical features of T2DM often include lipid disorders, characterized by the impact on small dense particles, including decreased circulating high-density lipoprotein cholesterol (HDL-C) levels (3). Thus, T2DM is closely linked to “metabolic syndrome” (4). Some scholars have pointed out that lipid traits can be used to predict the occurrence and progression of T2DM, and controlling blood lipids is also part of T2DM treatment (5, 6).

Since the 1970s, HDL-C has been referred to as “good cholesterol” in the field of cardiovascular diseases (CVD). However, its role has been reevaluated since the 21st century (7). Researchers studying HDL and CVD have found that increasing HDL-C levels is usually accompanied by effective blood glucose control in patients with T2DM (8, 9). This phenomenon has been confirmed in studies on cholesterol ester transfer protein (CETP) inhibitors (10). Some new perspectives have emerged, suggesting that HDL-C can reduce the risk of T2DM, and many prospective cohorts have confirmed this (9, 11). However, these conclusions are not consistent. For example, He et al. (12) believe that HDL-C does not reflect the risk of DM and needs to be combined with triglyceride (TG) levels for reference. The research conducted by Hwang et al. (13) indicates that an elevation in HDL-C is not significantly correlated with a reduced risk of developing T2DM within 2.8 years. Instead, it is associated with HDL-C/ApoA-I.

Due to the susceptibility of glucose and lipid metabolism to various factors, the association between HDL-C and diabetes remains controversial (3). Therefore, the purpose of this study is to explore the correlation between HDL-C levels and T2DM risk using cross-sectional data from the National Health and Nutrition Examination Survey (NHANES). Furthermore, although we have obtained some support from human and animal model studies that increasing HDL-C levels may help improve blood glucose control in patients with T2DM, it is currently uncertain whether this benefit is causally related to the increase in HDL-C levels. This is also a question that traditional observational studies find difficult to clarify. Therefore, this study will also incorporate Mendelian randomization (MR) analysis to verify the conclusions of NHANES from the perspective of genetic variation and further evaluate the causal relationship between the two. Mendelian randomization is an analytical method that explores how certain behaviors, environments, or other factors lead to specific health outcomes by utilizing the causal effects of human genetic variation on disease exposure (14). Due to the random segregation of the two alleles of single nucleotide polymorphisms (SNPs) according to Mendel’s law, Mendelian randomization has a natural advantage over traditional cohort study methods in being less susceptible to confounding factors (15). This will help clarify the causal effects of HDL-C levels on T2DM risk.

## Materials and methods

### Study population in NHANES

**Figure 1:**
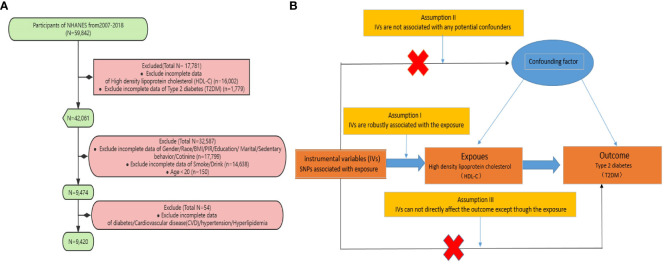
**(A)** Sampling flow during NHANES data analysis **(B)** Based on the three fundamental assumptions of Mendelian randomization, we evaluated the causal contribution of HDL-C to T2DM: (1) Assumption 1: Instrumental single nucleotide polymorphisms (SNPs) are strongly associated with HDL-C levels; (2) Assumption 2: The selected SNPs are independent of other confounding factors; (3) Assumption 3: The SNPs affect T2DM risk through their impact on HDL-C levels, without any other pathways involved.

The NHANES study is a multi-stage, stratified, and nationally representative study of the US population conducted by the National Center for Health Statistics of the Centers for Disease Control and Prevention. It aims to assess the nutrition and health status of Americans. The survey includes demographic, dietary, examination, laboratory, and questionnaire data. Prior to data collection, all study procedures were authorized by the ethics review committee of the National Center for Health Statistics, and informed consent forms were signed by all participants. For our study, we specifically analyzed data collected between 2007 and 2018. Subjects were excluded from our study for the following reasons: (1) Missing T2DM data; (2) Missing HDL-C data; (3) Aged under 20 years old; (4) Missing covariate (such as smoking, alcohol consumption, hypertension, hyperlipidemia, etc.) data (**Figure 1**).

We define T2DM as any of the following: (1) HbA1c levels equal to or greater than 6.5%; (2) serum glucose levels exceeding 200 mg/dL at 2 hours after a 75 g glucose load (OGTT); (3) fasting glucose levels equal to or greater than 126 mg/dL; (4) self-reported diagnosis of diabetes; (5) self-reported use of insulin or other diabetes medication. The duration of diabetes was determined by subtracting the participant’s current age from the self-reported age at diagnosis, or zero for individuals diagnosed during the NHANES examination.

The HDL-C data (Code: LBDHDDSI) included in our study, spanning from 2007 to 2018, were stored and analyzed by the University of Minnesota in Minneapolis, Minnesota, USA. Throughout this period, there were no changes in the laboratory methods or sites, but there were updates in the laboratory equipment. Specifically, from 2007 to 2012, the Roche Modular P Chemistry Analyzer was used for measuring the analyte, while from 2013 to 2018, the Roche Cobas 6000 and Roche Modular P Chemistry Analyzer were employed. The contracted laboratory adhered to the Westgard rules during the analysis of NHANES specimens (16), and the NHANES Quality Assurance and Quality Control (QA/QC) protocols complied with the requirements of the Clinical Laboratory Improvement Amendments of 1988, ensuring the reliability of the data source. Furthermore, several other common lipid traits, such as Low-density lipoprotein cholesterol (LDL-C) (code: LBDLDLSI), TG (code: LBDTRSI), and Total cholesterol (TC) (code: LBDTCSI), have been included in this study for further sensitivity analysis.

Our covariates included age, sex, race/ethnicity (Mexican American, other Hispanic, non-Hispanic white, non-Hispanic black, non-Hispanic Asian, other race), BMI, smoking status, alcohol consumption status, marital status, education level (less than 9th grade, 9-11 grade, high school graduate, some college/AA degree, and college graduate), poverty income ratio (PIR), cardiovascular disease, hypertension, and diabetes. BMI was divided into three categories: Normal (<25), Overweight (≥25, <30), and Obese (≥30). PIR was divided into three categories: Low (≤1.39), Medium (>1.39, <=3.49), and High (>3.49). Sedentary status is classified as severe (Defined as being sedentary for 480 minutes or more per day) and mild (Defined as being sedentary for less than 480 minutes per day) according to World Health Organization guidelines on physical activity levels (17). Smoking status was classified as Current Smoker (Defined as having smoked more than 100 cigarettes in a lifetime and still smoking), Former Smoker (Defined as having smoked more than 100 cigarettes in a lifetime but no longer smoking), Never smoke (defined as smoking less than 100 cigarettes in their lifetime). Alcohol status was classified as Current drinker (Defined as having consumed more than 12 alcoholic beverages of any type in a lifetime and still drinking), Former drinker (Defined as having consumed more than 12 alcoholic beverages of any type in a lifetime but not in the past year), Never drinker (Defined as not having had more than 12 alcoholic beverages of any type in a lifetime). Hypertension was defined according to the American Heart Association/American College of Cardiology (AHA/ACC) 2017 guidelines as systolic blood pressure≥130 mmHg or diastolic blood pressure ≥80 mmHg and self-reported diagnosis or use of antihypertensive medication. As per the guidelines set by the Adult Treatment Panel III of the National Cholesterol Education Program (NCEP-ATP III), hyperlipidemia is defined by the following criteria: total cholesterol levels equal to or exceeding 200mg/dL, triglyceride levels equal to or exceeding 150mg/dL, HDL cholesterol levels below 40mg/dL for men and below 50mg/dL for women, or LDL cholesterol levels equal to or exceeding 130mg/dL (18). Additionally, individuals who self-reported the use of cholesterol-lowering medications were also classified as having hyperlipidemia. For CVD, a positive response to any of the following questions was defined as CVD: “Has a doctor or other health professional ever told you that you have congestive heart failure (CHF)/coronary heart disease (CHD)/angina/heart attack/stroke?”.

### NHANES analysis

The NHANES database release files provide weights for each sample, such as the Mobile Examination Center (MEC) examination weights (19). The calculation of weights enables NHANES’ complex sampling design to achieve national representativeness. In this study, due to the inclusion of a large amount of laboratory data obtained in the MEC, we utilized the combined wtmec2yr for analysis. Additionally, since NHANES 2007-2018 combines data from 5 consecutive survey cycles (NHANES operates on a biennial cycle), the new weight (2007-2018) is calculated as 1/2 × wtmec2yr. The “survey” package’s svydesign function is employed for weighted computations. By utilizing quartiles, we can divide HDL-C levels into four categories (Q1, Q2, Q3, and Q4), with Q1 used as the reference. Continuous variables were reported as mean ± standard deviation (SD), while categorical variables were presented as individual counts (N) and percentages (%). Weighted t-tests (for continuous variables) or weighted chi-square tests (for categorical variables) were used to assess differences between T2DM and non-T2DM subjects. Kruskal-Wallis tests (for continuous variables) or weighted chi-square tests (for categorical variables) were used to evaluate differences among the four groups based on HDL-C exposure levels. Multivariable linear regression and restricted cubic splines (RCS) were used to analyze the correlation between HDL-C and T2DM. Initially, an unadjusted model was fitted, followed by stepwise adjustment for covariates. Model 1 adjusted for age, sex, and race; Model 2 further adjusted for PIR, BMI, marital, education level, cotinine, sedentary, alcohol, and smoking based on Model 1; Model 3 additionally adjusted for CVD, hyperlipidemia and hypertension based on Model 2. Results were presented as odds ratios (OR) with their corresponding 95% confidence intervals (95% CI). Subgroup analyses were conducted for significant results. Finally, a logistic regression model was used to assess the significance of the interaction between HDL-C and covariates on T2DM. In addition, considering the potential links of LDL-C, TC, and TG with DM, we categorized LDL-C, TC, and TG levels into three groups—Normal, Moderately elevated, and High - based on the recommendations for the general population outlined in the NCEP-ATP III. We conducted a sensitivity analysis to further adjust these lipids in the association between HDL-C and T2DM. Furthermore, we investigated the relationships between LDL-C, TC, TG, and T2DM. Finally, based on the above analyses, we conducted a reevaluation of subgroup analyses and interaction analyses.

### Mendelian randomized analysis

Under the framework of two-sample Mendelian randomization studies, this study utilizes SNPs as instrumental variables (IVs) for MR analysis, and adheres to the three fundamental assumptions of MR analysis (20). Assumption I posits that genetic variations are robustly associated with exposure (HDL-C). To minimize bias, it is important to use IVs with strong correlations (P< 5 × 10^-8^), and the F-value of the genetic tool should be above 10. Assumption II states that any confounding factors that influence the relationship between genetic variation and the outcome of exposure are not relevant. To address this issue, we restricted the sample population to Europeans. Additionally, we obtained phenotype information for each SNP from Phenoscanner (http://www.phenoscanner.medschl.cam.ac.uk/) and manually excluded any SNPs that were found to have an impact on outcome-related phenotypes. Assumption III asserts that the impact of genetic variation on the outcome can only be achieved through its association with the exposure. To address this issue, we utilized MR-Egger regression and the MR-PRESSO method to minimize the potential for horizontal pleiotropy and conducted sensitivity analyze.

### Selection of genetic instruments

To construct IVs for proxying HDL-C, we obtained summary statistics data from the Global Lipids Genetics Consortium (GLGC) GWAS, which included 94,595 individuals of European ancestry (21). The IVs for T2DM were obtained from a GWAS meta-analysis, which included a total of 12,931 T2DM patients and 57,196 healthy controls of European ancestry (22).

### Statistical power

We assessed the strength of the SNPs used as instruments using the F-statistic and considered only SNPs with an F-statistic greater than 10 to minimize weak instrumental bias (20). The instrument strength (F-statistic) was calculated using the following equation: F= [R^2^ (N-K-1)/K (1- R^2^)]. [N denotes the sample size of the exposure factor, k denotes the number of SNPs in each instrument, and R^2^ denotes the variance explained by the instrument. R^2^ = 2×EAF× (1-EAF) × Beta^2^, which was calculated from the equation proposed by Shim et al. (23)].

### Statistical analysis

We used a threshold of P < 5 × 10^-8^ and LD r^2^ ≤0.001 as the extraction criteria for exposure’ IVs. We employed the inverse variance weighting (IVW) method as our primary analysis, supplemented by weighted median, weighted mode, MR-Egger and simple mode. Due to the potential association of TC, TG, and LDL-C with T2DM, we constructed a multivariable Mendelian randomization (MVMR) model to eliminate their impact on the causal relationship between HDL-C and T2DM. MVMR is an extension of the standard univariate MR, designed to analyze the causal effects of different exposures on outcomes and estimate the direct causal effects of each exposure in a single analysis (24).

### Pleiotropy and Sensitivity analysis

We used MR-Egger regression to assess the possibility of horizontal pleiotropy, and the average pleiotropic effect of the IVs was represented by the intercept term of the MR-Egger regression (25). Additionally, the MR Pleiotropy REsidual Sum and Outlier (MR-PRESSO) were used as a supplement to evaluate horizontal pleiotropy. Its functionalities include detecting horizontal pleiotropy, correcting for horizontal pleiotropy by removing outliers, and determining if there are substantial changes in the causal effects before and after removing outliers (26). Heterogeneity was quantified using Cochran’s Q statistic. Moreover, a leave-one-out analysis was performed to assess the influence of each peripheral SNP on the results.

The statistical analyses for this study were conducted using R (version 4.2.2). Additionally, MR analysis utilized the R package “TwoSampleMR”. A two-tailed P-value < 0.05 was considered statistically significant.

## Results

### The baseline characteristics of the participants

Finally, a total of 9,420 individuals were included in this study. Based on the exclusion criteria, a total of 2,224 participants (18%) were classified as having T2DM. Compared to non- T2DM participants (7,196 individuals), T2DM patients generally tend to be older, have lower household income, engage in sedentary behavior more frequently, and exhibit a higher prevalence of obesity. Additionally, T2DM participants are more likely to have comorbidities such as hypertension, hyperlipidemia, and CVD. Finally, it is important to emphasize that compared to individuals without T2DM, those with T2DM exhibit a significant reduction in HDL-C levels. (**Table 1**) These findings highlight the distinct characteristics and health challenges faced by individuals with T2DM. According to the quartile method, we divided HDL-C levels into four intervals: Q1 (0.28-1.09], Q2 (1.09-1.32], Q3 (1.32-1.60], and Q4 (>1.60), with units in mmol/L. We observed that individuals with higher HDL-C levels had lower BMI, fewer smokers, and a lower prevalence of hypertension, hyperlipidemia, cardiovascular disease, and T2DM. On the other hand, individuals with lower HDL-C levels had a higher prevalence of higher PIR levels and alcohol consumption (**Supplementary Table S1**).

**Table 1:** Comparison between type2 diabetics and non-diabetics.

Characteristic	Overall, N = 9,420 (100%)^1,2^	Non-T2DM, N = 7,196 (82%)^2^	T2DM, N = 2,224 (18%)^2^	P Value^3^
Age (years) ***	49.2 (17.6)	46.8 (17.5)	60.2 (13.9)	**<0.001**
**Sex**				0.4
*Female*	5,968 (62%)	4,617 (62%)	1,351 (60%)	
*Male*	3,452 (38%)	2,579 (38%)	873 (40%)	
Race *				**0.010**
*Non-Hispanic White*	3,325 (61%)	2,614 (62%)	711 (58%)	
*Non-Hispanic Black*	2,148 (13%)	1,591 (12%)	557 (15%)	
*Other Race - Including Multi-Racial*	1,536 (11%)	1,211 (10.6%)	325 (11%)	
*Mexican American*	1,416 (9.1%)	1,035 (8.9%)	381 (9.8%)	
*Other Hispanic*	995 (6.5%)	745 (6.5%)	250 (6.2%)	
BMI (Kg/m^2^)***				**<0.001**
*Normal(<25)*	2,545 (27%)	2,252 (31%)	293 (11%)	
*Obese(≥30)*	3,943 (42%)	2,623 (37%)	1,320 (64%)	
*Overweight(≥25,<30)*	2,932 (31%)	2,321 (32%)	611 (25%)	
Education ***				**<0.001**
*9-11th Grade (Includes 12th grade with no diploma)*	1,259 (10.0%)	919 (9.5%)	340 (12%)	
*Less Than 9th Grade*	1,081 (6.1%)	674 (5.0%)	407 (11%)	
*High School Grad/GED or Equivalent*	2,795 (31%)	2,194 (31%)	601 (30%)	
*Some College or AA degree*	2,222 (26%)	1,703 (25%)	519 (27%)	
*College Graduate or above*	2,063 (27%)	1,706 (29%)	357 (19%)	
Marital ***				**<0.001**
*Divorced*	4,900 (56%)	3,688 (55%)	1,212 (60%)	
*Living with partner*	994 (7.6%)	633 (6.5%)	361 (13%)	
*Married*	993 (9.6%)	722 (9.3%)	271 (11%)	
*Never married*	304 (2.4%)	222 (2.3%)	82 (2.4%)	
*Separated*	1,569 (17%)	1,351 (19%)	218 (9.6%)	
*Widowed*	660 (7.1%)	580 (7.7%)	80 (4.5%)	
PIR **				**0.002**
*High(>3.49)*	2,469 (38%)	1,975 (39%)	494 (33%)	
*Low(≤1.39)*	3,434 (26%)	2,553 (26%)	881 (29%)	
*Medium(>1.39,<=3.49)*	3,517 (36%)	2,668 (35%)	849 (39%)	
Drinking.status ***				**<0.001**
*Current drinker*	4,002 (50%)	3,215 (52%)	787 (42%)	
*Former drinker*	2,158 (21%)	1,512 (20%)	646 (27%)	
*Never-drinker*	3,260 (29%)	2,469 (28%)	791 (31%)	
**Cotinine**	43 (114)	44 (114)	39 (115)	0.4
**Sedentary behavior** (Minutes/day) *				**0.048**
*Mild(<480)*	6,584 (67%)	5,058 (67%)	1,526 (64%)	
*Severe(≥480)*	2,836 (33%)	2,138 (33%)	698 (36%)	
Smoking.status ***				**<0.001**
*Current Smoker*	1,219 (13%)	988 (14%)	231 (10%)	
*Former Smoker*	1,721 (19%)	1,145 (17%)	576 (27%)	
*Never-Smoker*	6,480 (68%)	5,063 (69%)	1,417 (63%)	
Hypertension ***	4,358 (40%)	2,737 (33%)	1,621 (72%)	**<0.001**
Hyperlipidemia ***	6,856 (71%)	4,917 (67%)	1,939 (88%)	**<0.001**
CVD ***	1,125 (9.7%)	583 (6.6%)	542 (24%)	**<0.001**
HDL (mmol/L)***	1.37 (0.39)	1.40 (0.39)	1.23 (0.33)	**<0.001**

^1^Mean ± SD for continuous; n (%) for categorical. The percentages reported in this table are weighted statistics representing the proportions of the entire U.S. population. These values were obtained by performing weighted calculations using the “wtmec2yr” from the MEC (Mobile Examination Center) examination weights, utilizing the svydesign function from the “survey” package for weighted computation.

^2^t-test adapted to complex survey samples; chi-squared test with Rao & Scott’s second-order correction.

^3^*P < 0.05; **P < 0.01; ***P < 0.001.

T2DM, type 2 diabetes mellitus; BMI, Body mass index; PIR, poverty income ratio; CVD, cardiovascular diseases; HDL-C, high-density lipoprotein cholesterol.

### Associations between HDL-C and T2DM outcomes

**Figure 2:**
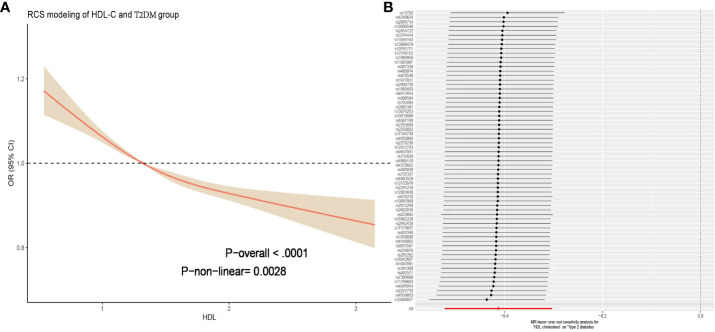
**(A)** Figure represents the relationship between HDL-C and T2DM adjusted for age; sex; race; education; marital; PIR; BMI; sedentary behavior; cotinine; alcohol; smoke; hypertension; hyperlipidemia; CVD. The solid red line represents the combined restricted cubic spline curve model, and the shaded area represents the 95% confidence interval of the combined curve. **(B)** The leave-one analysis in the two-sample Mendelian randomization analysis of HDL-C for T2DM mellitus showed that no SNP had a significant effect on the outcome (all rows were on the right side of 0). T2DM, type 2 diabetes mellitus; BMI, Body mass index; PIR, poverty income ratio; CVD, cardiovascular diseases; HDL-C, high-density lipoprotein cholesterol; SNP, single nucleotide polymorphism.

By constructing a multiple linear regression model, we found that when using Q1 (low HDL-C) as the reference, in all models, the Q2, Q3, and Q4 groups were significantly negatively associated with the risk of T2DM (P < 0.001). The risk of T2DM decreased as HDL-C levels increased, and this result remained robust after adjusting for covariates. Particularly, after adjusting for strict variables such as age, gender, race, education level, marital status, simvastatin, sedentary behavior, PIR, alcohol consumption, smoking, hypertension, hyperlipidemia and CVD (model 3), compared to the Q1 group, the average risk ratios for T2DM decreased by 23%, 49%, and a remarkable 71% in the Q2, Q3, and Q4 groups, respectively. These results did not differ significantly across different models, indicating the reliability of HDL-C in predicting T2DM risk (**Table 2**). We further utilized the restricted cubic spline (RCS) model to capture the non-linear relationship between HDL-C and T2DM. After adjusting for covariates, this relationship remained significant (P = 0.028, **Figure 2A**).

**Table 2:** Weighted multivariate adjusted logistic regression and subgroup analysis of T2DM risk with different HDL levels in NHANES from 2007 to 2018.

Regression model	Crude Model OR (95% CI)	Model 1 OR (95% CI)	Model 2 OR (95% CI)	Model3 OR (95% CI)
HDL-C (mmol/L)				
Q1(0.28-1.09]	Reference	Reference	Reference	Reference
Q2(1.09-1.32]	0.66 (0.57,0.77) ***	0.56 (0.47,0.66) ***	0.60 (0.51,0.71) ***	0.67 (0.57,0.79) ***
Q3(1.32-1.60]	0.47 (0.40,0.57) ***	0.34 (0.27,0.42) ***	0.41 (0.33,0.51) ***	0.51 (0.40,0.65) ***
Q4 (>1.60)	0.27 (0.22,0.32) ***	0.16 (0.13,0.20) ***	0.24 (0.20,0.30) ***	0.29 (0.23,0.36) ***
Subgroup	HDL-C—Q2(1.09-1.32] OR(95%CI)-P Value	HDL-C—Q3 (1.32-1.60] OR(95%CI)-P Value	HDL-C—Q4(>1.60) OR(95%CI)-P Value	Interaction P Value
**Age**				P=0.006 **
*20-30*	0.65 (0.22, 1.92) P=0.4	0.86 (0.21, 3.56) P=0.8	0.70 (0.12, 3.97) P=0.7	
*31-45*	0.55 (0.37, 0.82) P=0.004	0.79 (0.32, 1.92) P=0.6	0.48 (0.25, 0.90) P=0.022	
*46-60*	0.72 (0.47, 1.09) P=0.12	0.53 (0.33, 0.85) P=0.009	0.27 (0.16, 0.45) P<0.001	
*>60*	0.60 (0.46, 0.79) P<0.001	0.42 (0.31, 0.59) P<0.001	0.27 (0.20, 0.36) P<0.001	
**Race**				P=0.089
*Non-Hispanic White*	0.58 (0.34, 0.99) P=0.048	0.37 (0.21, 0.63) P<0.001	0.31 (0.17, 0.54) P<0.001	
*Non-Hispanic Black*	0.69 (0.45, 1.07) P=0.094	0.46 (0.29, 0.72) P=0.001	0.34 (0.22, 0.53) P<0.001	
*Other Race - Including Multi-Racial*	0.58 (0.35, 0.94) P=0.03	0.78 (0.38, 1.59) P=0.5	0.32 (0.17, 0.62) P=0.001	
*Mexican American*	0.63 (0.50, 0.81) P<0.001	0.50 (0.36, 0.69) P<0.001	0.24 (0.16, 0.34) P<0.001	
*Other Hispanic*	0.91 (0.58, 1.41) P=0.7	0.75 (0.44, 1.26) P=0.3	0.40 (0.23, 0.70) P=0.002	
**BMI (Kg/m^2^)**				P=0.502
*Normal(<25)*	0.65 (0.52, 0.81) P<0.001	0.48 (0.33, 0.70) P<0.001	0.24 (0.17, 0.34) P<0.001	
*Obese(≥30)*	0.67 (0.46, 0.96) P=0.03	0.59 (0.42, 0.81) P=0.002	0.31 (0.21, 0.46) P<0.001	
*Overweight(≥25,<30)*	0.68 (0.42, 1.12) P=0.13	0.38 (0.22, 0.66) P<0.001	0.36 (0.19, 0.69) P=0.003	
**PIR**				P=0.363
*High(>3.49)*	0.57 (0.40, 0.80) P=0.002	0.39 (0.26, 0.60) P<0.001	0.23 (0.14, 0.36) P<0.001	
*Low(≤1.39)*	0.71 (0.52, 0.97) P=0.031	0.49 (0.35, 0.70) P<0.001	0.36 (0.25, 0.52) P<0.001	
*Medium(>1.39,<=3.49)*	0.69 (0.51, 0.94) P=0.018	0.60 (0.38, 0.93) P=0.023	0.28 (0.19, 0.42) P<0.001	
**Sedentary.status(Minutes/day)**				P=0.295
*Mild(<480)*	0.71 (0.58, 0.88) P=0.002	0.54 (0.40, 0.73) P<0.001	0.35 (0.28, 0.45) P<0.001	
*Severe(≥480)*	0.58 (0.42, 0.80) P=0.001	0.45 (0.28, 0.75) P=0.002	0.19 (0.12, 0.31) P<0.001	
**Drinking. status**				P=0.059
*Current drinker*	0.51 (0.42, 0.62) P<0.001	0.47 (0.34, 0.65) P<0.001	0.21 (0.15, 0.29) P<0.001	
*Former drinker*	0.67 (0.46, 0.97) P=0.035	0.59 (0.39, 0.89) P=0.013	0.30 (0.20, 0.44) P<0.001	
*Non-drinker*	0.91 (0.65, 1.27) P=0.6	0.47 (0.30, 0.73) P=0.001	0.42 (0.29, 0.62) P<0.001	
**Smoking. status**				P=0.231
*Current Smoker*	0.49 (0.28, 0.84) P=0.011	0.34 (0.14, 0.82) P=0.018	0.34 (0.15, 0.74) P=0.007	
*Former Smoker*	0.52 (0.36, 0.76) P=0.001	0.39 (0.25, 0.59) P<0.001	0.17 (0.11, 0.26) P<0.001	
*Non-Smoker*	0.80 (0.62, 1.04) P=0.091	0.63 (0.47, 0.84) P=0.002	0.36 (0.27, 0.47) P<0.001	
**Hypertension**				P=0.564
*Yes*	0.67 (0.47, 0.95) P=0.024	0.55 (0.37, 0.81) P=0.003	0.35 (0.20, 0.59) P<0.001	
*No*	0.69 (0.56, 0.85) P<0.001	0.51 (0.37, 0.69) P<0.001	0.27 (0.21, 0.34) P<0.001	
**Hyperlipidemia**				P=0.463
*Yes*	0.78 (0.39, 1.57) P=0.5	0.62 (0.28, 1.38) P=0.2	0.43 (0.18, 1.04) P=0.06	
*No*	0.67 (0.55, 0.80) P<0.001	0.50 (0.38, 0.65) P<0.001	0.27 (0.21, 0.35) P<0.001	
**CVD**				P=0.952
*Yes*	0.66 (0.53, 0.81) P<0.001	0.50 (0.38, 0.66) P<0.001	0.27 (0.21, 0.35) P<0.001	
*No*	0.76 (0.48, 1.20) P=0.2	0.58 (0.31, 1.05) P=0.072	0.39 (0.24, 0.61) P<0.001	

*P < 0.05; **P < 0.01; ***P < 0.001.

Multiple logistic regression model: Model 1: Adjusted for Age; Sex; Race; Model 2: Adjusted for Age; Sex; Race; Education; Marital; PIR; BMI; Sedentary behavior; Cotinine; Alcohol; Smoke; Model 3: Adjusted for Age; Sex; Race; Education; Marital; PIR; BMI; Sedentary behavior; Cotinine; Alcohol; Smoke; Hypertension; Hyperlipidemia; CVD.

Subgroup Analysis Adjustment Factors: Age; Sex; Race; Education; Marital status; PIR; Body Mass Index; Sedentary behavior; Simvastatin; Alcohol; Smoking; Hypertension; Hyperlipidemia; CVD, excluding sub-group variables, and the reference object in the sub-group is Q1 of HDL-C.

T2DM, type 2 diabetes mellitus; BMI, Body mass index; PIR, poverty income ratio; CVD, cardiovascular diseases; HDL-C, high-density lipoprotein cholesterol.

We conducted interaction analyses for the covariates that showed significant differences between T2DM and non-T2DM in **Table 1**. The results showed that the effect of HDL-C levels on T2DM varied with age (P for interaction = 0.006, **Table 2**), while there were no significant interactions for the other covariates (P > 0.05). Using the Q1 range of HDL-C levels as the reference, we subdivided age into young (20-30], middle-aged (31-45], middle-old (46-60], and elderly (>60) groups (while adjusting for other covariates). We found that there was no association between HDL-C and T2DM in the young population, but as age increased, this association strengthened. Particularly for the elderly group, individuals in the Q2 range already had a 40% lower risk of T2DM [OR=0.60, 95%CI (0.46, 0.79), P < 0.001], and the Q4 range had a 73% lower risk [OR=0.27, 95%CI (0.20, 0.36), P < 0.001]. HDL-C levels showed varying degrees of association with reducing T2DM risk in other subgroups.

In the further sensitivity analysis, we conducted a reanalysis after including the additional variables LDL-C, TC, and TG (baseline characteristics of patients are provided in **Supplementary Table S2**). The results showed that despite changes in the included population, the association between HDL-C in the Q4 group (>1.60) and T2DM remained robust [OR=0.43, 95%CI (0.20, 0.92), P = 0.03]. In subgroup analysis results, there was no interaction between other lipid levels and the reduction of T2DM risk by HDL-C, demonstrating the reliability of HDL-C in predicting T2DM. (**Supplementary Table S3**) Furthermore, after adjusting for age, sex, race, education, marital status, PIR, BMI, sedentary behavior, cotinine, alcohol, smoking, hypertension, hyperlipidemia, CVD, LDL-C, HDL-C, TC, and TG separately (with lipid excluded as independent variable), LDL-C, TC, and TG were no longer statistically associated with T2DM. (**Supplementary Table S4**).

### MR estimates

When using HDL-C as an exposure instrument, the assessment of genetic instrument strength, as indicated by a Total-F-statistic of 106.94 (R^2^ = 4%, with individual SNP F-values ranging from 29.95 to 962.62, **Supplementary Table S5**), suggests that there is no evidence of statistical bias resulting from weak instrument bias. In the IVW analysis, we observed a significant inverse association between HDL-C levels and the risk of T2DM (OR = 0.69, 95% CI = 0.52-0.82; P = 1.41× 10^-13^). This finding indicates that for per 1 mmol/L increase in HDL-C. All alternative analytical approaches yielded statistically significant results that were consistent with the IVW analysis. Moreover, no evidence of heterogeneity or horizontal pleiotropy was observed, as indicated by non-significant Cochran’s Q test and MR-Egger intercept/MR-Presso p-values >0.05 (**Table 3**). Additionally, a sensitivity analysis (leave-one-out analysis) demonstrated the stability of the results, showing consistent findings even when individual SNPs were excluded (**Figure 2B**). These findings provide robust statistical evidence supporting the negative association between HDL-C levels and the risk of T2DM. The MVMR analysis, adjusted for LDL-C, TC and TG, showed that HDL-C results for T2DM remained robust. (P =8.02× 10^-3^, **Supplementary Table S6**). The consistency across different analytical methods strengthens the validity and reliability of our study’s conclusions. The results of the reverse MR analysis elucidated that having T2DM is not causally associated with HDL-C levels [IVW, OR: 1.00, 95% CI (0.97, 1.03), P = 0.089]. Additionally, the heterogeneity, horizontal pleiotropy and sensitivity analysis of the reverse MR analysis was also deemed reliable (**Supplementary Table S7**).

**Table 3:** Causal association and sensitivity analysis between HDL-C and T2DM in two-sample Mendelian randomization analysis.

Exposure	Outcome	SNP	Methods	OR (95% CI)	P.value
High density lipoprotein cholesterol (HDL-C)	Type 2 diabetes mellitus (T2DM)	65	MR Egger	0.78(0.63,0.96)	0.025
			Weighted median	0.69(0.58,0.82)	3.45× 10^-5^
			Inverse variance weighted	0.66(0.59,0.74)	1.41× 10^-13^
			Simple mode	0.61(0.44,0.85)	4.48× 10^-3^
			Weighted mode	0.70(0.58,0.84)	3.04× 10^-4^
			**Cochran Q- P.value**	**MR-Egger Intercept-P.value**	**MR-PRESSO- P.value**
			0.861	0.076	0.447

The MR-Egger Intercept and MR-PRESSO P-values in the table are used to investigate the presence of horizontal pleiotropy. A P-value > 0.05 indicates the absence of horizontal pleiotropy, suggesting that the study aligns with the basic assumptions of Mendelian randomization. On the other hand, the P-value of Cochran’s Q test explores the presence of heterogeneity. A P-value > 0.05 indicates no significant heterogeneity, indicating an association between the instrumental variables and phenotype.

## Discussion

With further research on HDL, several additional functional characteristics have been discovered, including cholesterol efflux capacity (CEC) (27), antioxidant properties (28) and anti-inflammatory effects (29). HDL is considered to have an anti-diabetic effect for the following reasons:

Firstly, *in vitro* studies have shown that HDL-C activates adenosine monophosphate-activated protein kinase (AMPK) (8), an energy-sensing enzyme, which increases ATP production and glucose uptake in skeletal muscles (30), thereby promoting glycogen synthesis in response to decreased insulin levels (31). Disruption of cholesterol homeostasis in pancreatic β-cells can impair insulin secretion (32), while HDL’s CEC, which involves transporting excess cholesterol from tissues to the liver for metabolism and excretion, helps maintain lipid balance in cells (33). HDL’s anti-inflammatory effects also play a role in preventing T2DM. Inflammation factors in the body and adipose tissue play an essential role in inducing insulin resistance, a key factor in the progression of T2DM (34). Therefore, reducing inflammation has been considered an effective therapeutic method to potentially improve insulin sensitivity (35). HDL inhibits inflammation by reducing the activation of NF-κB in endothelial cells, activating the cytoprotective enzyme heme oxygenase-1, and inhibiting inflammasome activation (36, 37). HDL also inhibits the pro-inflammatory effects of macrophages, which is crucial for preventing the progression of atherosclerotic lesions (37, 38). Oxidative stress is both a significant cause and result of T2DM. Reactive oxygen species can directly damage pancreatic β-cells, interfere with the normal functioning of insulin signaling pathways, and reduce cellular responsiveness to insulin, leading to elevated blood glucose levels (39). HDL-related antioxidant enzyme paraoxonases-1 (PON1) can enhance the clearance of lipid hydroperoxides, thereby reducing oxidative stress. Experimental evidence has shown that this can increase insulin secretion in mouse and cell models (40).

It is well known that the progression of T2DM is driven by a decrease in the mass and function of pancreatic β-cells. The number of β-cells naturally decreases in the human body as a result of age-related factors such as cell apoptosis, impaired cell function, and a decline in pancreatic regenerative capacity. HDL can inhibit β-cell apoptosis (41, 42) and protect β-cells from oxidative damage caused by low-density lipoprotein (LDL) oxidation (28), thereby improving β-cell survival. The results of this study also indicate a significant interaction between age and the association between HDL-C and T2DM. Specifically, the risk of T2DM in young individuals is not strongly associated with HDL-C levels, possibly due to the better regenerative function of β-cells in young people. However, as age increases, the risk of T2DM in humans may be more influenced by HDL levels, especially in individuals aged 61 and above. Compared to individuals with low HDL-C levels (HDL ≤ 1.09 mmol/L) of the same age, those with HDL-C levels greater than 1.60 mmol/L can reduce their T2DM risk by 73%, and the result remained robust after adjusting for other lipid variables (LDL-C, TC and TG). In fact, this is consistent with the changing pattern of HDL-C levels throughout a person’s life. Although data on the age-related changes in HDL-C are contradictory, prospective studies have shown that adult HDL-C tends to decrease with age (43). In the mechanism of species-specific aging, replicative aging caused by telomere shortening leads to the loss of cellular division capacity. This process is a significant factor that may result in a decrease in both the concentration and functionality of HDL-C (44). Additionally, the decline in hormone levels (testosterone, sex hormone-binding globulin, etc.) with age is also an important factor in the decrease of HDL (45, 46). Studies have shown that compared to Korean women in their 20s, those in their 70s have a decrease in HDL-C levels by up to 8.15 mg/dL (approximately 0.21 mmol/L) (47), and this downward trend continues with age (48).

Our study has the following strengths: 1.This is the first cross-sectional study using the NHANES database to analyze a cohort of over 9,000 subjects over a period of 12 years, establishing HDL-C as an independent factor in preventing T2DM. 2. Our study adjusted for various covariates and demonstrated that HDL-C levels above 1.60 mmol/L have a strong capacity to reduce the risk of T2DM. We also conducted age-based subgroup analyses, indicating that older adults should pay particular attention to their HDL-C levels. This finding strengthens its clinical significance. 3. For the first time, we used a combination of NHANES database and MR methods to explore the relationship between HDL-C and T2DM at both clinical and genetic levels. Our analysis followed the three fundamental assumptions of Mendelian randomization, allowing for causal inference, and reverse MR analysis further confirmed our findings.

However, this study also has certain limitations: Firstly, the imprecise measurement of HDL-C levels and the retrospective diagnosis of T2DM based on questionnaires may affect the estimation of the association between HDL-C and T2DM risk in a cross-sectional study. Therefore, we conducted a 2-sample Mendelian randomization (2SMR) study to further elucidate the causal relationship. Secondly, considering that the NHANES and 2SMR data mostly come from non-Hispanic white and European ancestry participants, it remains unclear whether the same results can be applied to other racial/ethnic groups. However, the cross-sectional analysis in NHANES indicated no interaction between race/ethnicity and the association between HDL-C and T2DM (P for interaction = 0.089), and subgroup and sensitivity analysis demonstrated no significant impact of race/ethnicity on the results (**Table 2**). This suggests that race/ethnicity did not play a major role in this study. Finally, we observed that Huang et al. (49) also investigated the relationship between T2DM and HDL-C. The differences in our conclusions may stem from: 1. Differences in the selection of data sources for T2DM, 2. we noted a distinction in the original GWAS data on T2DM (50) regarding adjusted and unadjusted BMI, which Huang M’s paper did not further clarify. Whether BMI was adjusted could also potentially impact the study results, and in our research, we excluded BMI-related SNPs. We fully agree with Huang et al.’s suggestion that insulin resistance could influence the association between T2DM and HDL-C, which warrants attention in future research.

## Conclusion

Our study suggests a causal relationship between increasing HDL-C levels and reducing the risk of T2DM, with a stronger effect observed in older individuals. In the future, it is necessary to further elucidate the underlying mechanisms and validate whether interventions targeting HDL-C levels can indeed lower the risk of T2DM.

## Data availability statement

The original contributions presented in the study are included in the article/**Supplementary Material**. Further inquiries can be directed to the corresponding authors.

## Author contributions

ZY: Methodology, Software, Writing – original draft. YX: Conceptualization, Methodology, Software, Writing – original draft. KL: Methodology, Software, Writing – original draft. LL: Writing – review & editing.
