# Supplementary material for: Association between high-density lipoprotein cholesterol and type 2 diabetes mellitus: dual evidence from NHANES database and Mendelian randomization analysis
Source: Front Endocrinol (Lausanne). 2024 Feb 22;15:1272314. doi: 10.3389/fendo.2024.1272314 (PMC10917910; doi:10.3389/fendo.2024.1272314)
Supplement: Supplementary file 1 [file Table_1.docx]

**Table S1. Participant characteristics by HDL-C quartile interval (NHANES 2007-2018 N = 9,420)**

| **Characteristic** | **Overall**, N = 9420 (100%)^1,2^ | **Q1**, N = 2498 (26%)^2^ | **Q2**, N = 2440 (26%)^2^ | **Q3**, N = 2277 (24%)^2^ | **Q4**, N = 2205 (24%)^2^ | **P Value**^3^ |
| --- | --- | --- | --- | --- | --- | --- |
| **Age (years)** | 49.2 | 47.7 | 48.3 | 49.8 | 51.3 |  |
| **Sex ***** |  |  |  |  |  | **<0.001** |
| *Female* | 5,968 (62%) | 1,043 (38%) | 1,449 (56%) | 1,656 (72%) | 1,820 (83%) |  |
| *Male* | 3,452 (38%) | 1,455 (62%) | 991 (44%) | 621 (28%) | 385 (17%) |  |
| **Race ***** |  |  |  |  |  | **<0.001** |
| *Non-Hispanic White* | 3,325 (61%) | 998 (62%) | 829 (61%) | 748 (59%) | 750 (63%) |  |
| *Non-Hispanic Black* | 2,148 (13%) | 410 (9.3%) | 530 (12%) | 592 (14%) | 616 (15%) |  |
| *Other Race - Including Multi-Racial* | 1,536 (11%) | 380 (10.1%) | 389 (10.3%) | 370 (11.9%) | 397 (10.2%) |  |
| *Mexican American* | 1,416 (9.1%) | 426 (11%) | 408 (9.9%) | 335 (9.0%) | 247 (6.5%) |  |
| *Other Hispanic* | 995 (6.5%) | 284 (7.6%) | 284 (6.8%) | 232 (6.1%) | 195 (5.3%) |  |
| **BMI ***** |  |  |  |  |  | **<0.001** |
| *Normal(<25)* | 2,545 (27%) | 372 (14%) | 511 (20%) | 695 (31%) | 967 (46%) |  |
| *Obese(≥30)* | 3,943 (42%) | 1,327 (55%) | 1,150 (48%) | 869 (38%) | 597 (25%) |  |
| *Overweight(≥25,<30)* | 2,932 (31%) | 799 (31%) | 779 (32%) | 713 (31%) | 641 (29%) |  |
| **Education ***** |  |  |  |  |  | **<0.001** |
| *9-11th Grade (Includes 12th grade with no diploma)* | 1,259 (10.0%) | 373 (12%) | 341 (11%) | 304 (9.8%) | 241 (7.5%) |  |
| *Less Than 9th Grade* | 1,081 (6.1%) | 315 (6.8%) | 310 (6.9%) | 256 (5.8%) | 200 (4.8%) |  |
| *Non-Hispanic Black* | 2,795 (31%) | 719 (30%) | 725 (32%) | 676 (30%) | 675 (31%) |  |
| *Non-Hispanic White* | 2,222 (26%) | 648 (30%) | 578 (26%) | 527 (26%) | 469 (21%) |  |
| *Other Race - Including Multi-Racial* | 2,063 (27%) | 443 (22%) | 486 (24%) | 514 (29%) | 620 (36%) |  |
| **Marital **** |  |  |  |  |  | **0.004** |
| *Divorced* | 4,900 (56%) | 1,381 (59%) | 1,305 (56%) | 1,138 (55%) | 1,076 (54%) |  |
| *Living with partner* | 994 (7.6%) | 200 (5.6%) | 234 (6.8%) | 244 (8.4%) | 316 (10%) |  |
| *Married* | 993 (9.6%) | 233 (8.0%) | 231 (9.3%) | 261 (10%) | 268 (11%) |  |
| *Never married* | 304 (2.4%) | 73 (2.4%) | 84 (2.7%) | 78 (2.3%) | 69 (2.0%) |  |
| *Separated* | 1,569 (17%) | 426 (18%) | 400 (18%) | 403 (17%) | 340 (16%) |  |
| *Widowed* | 660 (7.1%) | 185 (7.0%) | 186 (7.6%) | 153 (6.9%) | 136 (6.9%) |  |
| **PIR ***** |  |  |  |  |  | **<0.001** |
| *High(>3.49)* | 2,469 (38%) | 552 (33%) | 608 (37%) | 618 (39%) | 691 (43%) |  |
| *Low(≤1.39)* | 3,434 (26%) | 1,015 (30%) | 918 (28%) | 819 (26%) | 682 (21%) |  |
| *Medium(>1.39,<=3.49)* | 3,517 (36%) | 931 (38%) | 914 (36%) | 840 (35%) | 832 (35%) |  |
| **Drinking.status ***** |  |  |  |  |  | **<0.001** |
| *Current drinker* | 4,002 (50%) | 1,019 (46%) | 1,007 (50%) | 968 (51%) | 1,008 (55%) |  |
| *Former drinker* | 2,158 (21%) | 644 (25%) | 542 (20%) | 521 (21%) | 451 (18%) |  |
| *Never-drinker* | 3,260 (29%) | 835 (29%) | 891 (29%) | 788 (29%) | 746 (27%) |  |
| **Sedentary behavior** (**Minutes /day)** |  |  |  |  |  | 0.9 |
| *Mild(<480)* | 6,584 (67%) | 1,692 (66%) | 1,734 (67%) | 1,575 (67%) | 1,583 (67%) |  |
| *Severe(≥480)* | 2,836 (33%) | 806 (34%) | 706 (33%) | 702 (33%) | 622 (33%) |  |
| **Smoking.status ***** |  |  |  |  |  | **<0.001** |
| *Current Smoker* | 1,219 (13%) | 417 (17%) | 331 (13%) | 269 (13%) | 202 (9.0%) |  |
| *Former Smoker* | 1,721 (19%) | 526 (21%) | 470 (21%) | 376 (17%) | 349 (18%) |  |
| *Never- Smoker* | 6,480 (68%) | 1,555 (62%) | 1,639 (66%) | 1,632 (71%) | 1,654 (73%) |  |
| **Hypertension ***** | 4,358 (40%) | 1,228 (46%) | 1,162 (41%) | 990 (37%) | 978 (37%) | **<0.001** |
| **Hyperlipidemia ***** | 6,856 (71%) | 2,315 (91%) | 1,876 (75%) | 1,254 (55%) | 1,411 (60%) | **<0.001** |
| **CVD ***** | 1,125 (9.7%) | 365 (14%) | 303 (8.9%) | 241 (8.4%) | 216 (7.4%) | **<0.001** |
| **Diabetes ***** |  |  |  |  |  | **<0.001** |
| *T2DM* | 2,224 (18%) | 814 (28%) | 643 (20%) | 452 (15%) | 315 (9.2%) |  |
| *Non-T2DM* | 7,196 (82%) | 1,684 (72%) | 1,797 (80%) | 1,825 (85%) | 1,890 (91%) |  |

1Mean ± SD for continuous; n (%) for categorical. The percentages reported in this table are weighted statistics representing the proportions of the entire U.S. population. These values were obtained by performing weighted calculations using the "wtmec2yr" from the MEC examination weights, utilizing the svydesign function from the "survey" package for weighted computation

2t-test adapted to complex survey samples; chi-squared test with Rao & Scott's second-order correction

3*P < 0.05; **P < 0.01; ***P < 0.001

T2DM: type 2 diabetes mellitus; BMI: Body mass index; PIR: poverty income ratio; CVD: cardiovascular diseases; HDL-C: high-density lipoprotein cholesterol; EMC: Mobile Examination Center.

**Table S2. Comparison between type2 diabetics and non-diabetics (Adjustment based on other lipid traits)**

| **Characteristic** | **Overall**, N = 4150 (100%)^1,2^ | **No**, N = 3050 (80%)^2^ | **Yes**, N = 1100 (20%)^2^ | **P Value**^3^ |
| --- | --- | --- | --- | --- |
| **Age (years) ***** | 49.1 (17.5) | 46.1 (17.2) | 60.5 (13.6) | **<0.001** |
| **Sex** |  |  |  | 0.3 |
| *female* | 2,653 (62%) | 1,998 (62%) | 655 (60%) |  |
| *male* | 1,497 (38%) | 1,052 (38%) | 445 (40%) |  |
| **Race** |  |  |  | 0.14 |
| *Non-Hispanic White* | 1,473 (62%) | 1,113 (63%) | 360 (59%) |  |
| *Non-Hispanic Black* | 933 (12%) | 670 (12%) | 263 (14%) |  |
| *Other Race - Including Multi-Racial* | 676 (10%) | 523 (10%) | 153 (9.6%) |  |
| *Mexican American* | 642 (9.3%) | 438 (8.9%) | 204 (11%) |  |
| *Other Hispanic* | 426 (6.1%) | 306 (6.0%) | 120 (6.4%) |  |
| **BMI***** |  |  |  | **<0.001** |
| *Normal(<25)* | 1,185 (30%) | 1,039 (35%) | 146 (12%) |  |
| *Obese(≥30)* | 1,685 (40%) | 1,033 (34%) | 652 (64%) |  |
| *Overweight(≥25,<30)* | 1,280 (30%) | 978 (31%) | 302 (24%) |  |
| **PIR***** |  |  |  | **0.001** |
| *High(>3.49)* | 1,103 (37%) | 846 (39%) | 257 (31%) |  |
| *Low(≤1.39)* | 1,445 (25%) | 1,045 (25%) | 400 (27%) |  |
| *Medium(>1.39,<=3.49)* | 1,602 (37%) | 1,159 (36%) | 443 (41%) |  |
| **Education***** |  |  |  | **<0.001** |
| *9-11th Grade (Includes 12th grade with no diploma)* | 567 (10%) | 388 (9.4%) | 179 (13%) |  |
| *Less Than 9th Grade* | 453 (5.8%) | 278 (4.9%) | 175 (9.2%) |  |
| *Non-Hispanic Black* | 1,224 (31%) | 932 (31%) | 292 (28%) |  |
| *Non-Hispanic White* | 974 (26%) | 699 (25%) | 275 (30%) |  |
| *Other Race - Including Multi-Racial* | 932 (28%) | 753 (30%) | 179 (20%) |  |
| **Marital***** |  |  |  | **<0.001** |
| *Divorced* | 2,168 (56%) | 1,554 (55%) | 614 (59%) |  |
| *Living with partner* | 425 (7.0%) | 258 (5.9%) | 167 (11%) |  |
| *Married* | 439 (10%) | 302 (9.9%) | 137 (12%) |  |
| *Never married* | 139 (2.3%) | 94 (2.2%) | 45 (2.7%) |  |
| *Separated* | 688 (17%) | 593 (19%) | 95 (9.8%) |  |
| *Widowed* | 291 (7.4%) | 249 (8.0%) | 42 (4.9%) |  |
| **Drinking.status***** |  |  |  | **<0.001** |
| *Current drinker* | 1,767 (50%) | 1,369 (53%) | 398 (39%) |  |
| *Former drinker* | 985 (22%) | 654 (20%) | 331 (29%) |  |
| *Non-drinker* | 1,398 (28%) | 1,027 (27%) | 371 (32%) |  |
| **Smoking.status***** |  |  |  | **<0.001** |
| *Current* | 534 (13%) | 424 (14%) | 110 (9.7%) |  |
| *Former* | 746 (19%) | 461 (17%) | 285 (27%) |  |
| *Never* | 2,870 (68%) | 2,165 (69%) | 705 (63%) |  |
| **Cotinine** | 42 (114) | 44 (115) | 38 (108) | 0.5 |
| **Sedentary behavior (Minutes /day)** |  |  |  | 0.2 |
| *mild(<480)* | 2,897 (66%) | 2,149 (67%) | 748 (63%) |  |
| *severe(≥480)* | 1,253 (34%) | 901 (33%) | 352 (37%) |  |
| **Hypertension***** | 1,920 (40%) | 1,119 (32%) | 801 (71%) | **<0.001** |
| **Hyperlipidemia***** | 2,964 (69%) | 2,014 (64%) | 950 (88%) | **<0.001** |
| **CVD***** | 513 (9.8%) | 241 (6.3%) | 272 (23%) | **<0.001** |
| **HDL(mmol/L)***** | 1.41 (0.39) | 1.44 (0.39) | 1.26 (0.32) | **<0.001** |
| **LDL(mmol/L)***** | 2.89 (0.93) | 2.95 (0.90) | 2.65 (1.02) | **<0.001** |
| **TC(mmol/L)***** | 4.87 (1.07) | 4.93 (1.03) | 4.63 (1.16) | **<0.001** |
| **TG(mmol/L)***** | 1.24 (0.71) | 1.15 (0.66) | 1.58 (0.78) | **<0.001** |

1Mean ± SD for continuous; n (%) for categorical. The percentages reported in this table are weighted statistics representing the proportions of the entire U.S. population. These values were obtained by performing weighted calculations using the "wtmec2yr" from the MEC (Mobile Examination Center) examination weights, utilizing the svydesign function from the "survey" package for weighted computation

2t-test adapted to complex survey samples; chi-squared test with Rao & Scott's second-order correction.

LDL: Low-density lipoprotein; TG: Triglycerides; TC: Total cholesterol; HDL-C: high-density lipoprotein cholesterol; T2DM: type 2 diabetes mellitus; BMI: Body mass index; PIR: poverty income ratio; CVD: cardiovascular diseases; EMC: Mobile Examination Center.

3*P < 0.05; **P < 0.01; ***P < 0.001.

**Table S3. Weighted multivariate adjusted logistic regression and subgroup analysis of T2DM risk with different HDL-C levels (Adjustment based on other lipid traits)**

| **Regression model** | **Crude Model**  **OR (95% CI)** | **Model 1**  **OR (95% CI)** | **Model 2**  **OR (95% CI)** | **Model3**  **OR (95% CI)** |
| --- | --- | --- | --- | --- |
| **HDL-C (mmol/L)** |  |  |  |  |
| Q1(0.28-1.09] | Reference | Reference | Reference | Reference |
| Q2(1.09-1.32] | 0.62(0.48, 0.79)*** | 0.49(0.37, 0.67)*** | 0.73(0.50, 1.08) | 0.76(0.52, 1.11) |
| Q3(1.32-1.60] | 0.46(0.35, 0.60)*** | 0.29(0.21, 0.41) *** | 0.62(0.36, 1.07) | 0.7(0.40, 1.21) |
| Q4 (＞1.60) | 0.24(0.18, 0.32)*** | 0.12(0.09, 0.18) *** | 0.42(0.20, 0.88)* | 0.43(0.20, 0.92)* |
| **Subgroup** | **HDL-C—Q2(1.09-1.32]**  **OR(95%CI)-P Value** | **HDL-C—Q3 (1.32-1.60]**  **OR(95%CI)-P Value** | **HDL-C—Q4(＞1.60)**  **OR(95%CI)-P Value** | **Interaction P Value** |
| **Age** |  |  |  | P=0.042 |
| *20-30* | 3.06(0.47, 20.0)P=0.2 | 3.9(0.35, 43.2)P=0.3 | 4.21(0.23, 78.3)P=0.6 |  |
| *31-45* | 0.46(0.21, 1.00)P=0.049 | 0.41(0.18, 0.97)P=0.042 | 0.2(0.09, 0.47)P<0.001 |  |
| *46-60* | 0.68(0.39, 1.20)P=0.2 | 0.5(0.26, 0.94)P=0.033 | 0.2(0.10, 0.42)P<0.001 |  |
| *＞60* | 0.39(0.25, 0.62)P<0.001 | 0.32(0.19, 0.52)P<0.001 | 0.18(0.11, 0.29)P<0.001 |  |
| **Race** |  |  |  | P=0.177 |
| *Non-Hispanic White* | 0.79(0.34, 1.84)P=0.6 | 0.41(0.18, 0.96)P=0.041 | 0.44(0.17, 1.11)P=0.08 |  |
| *Non-Hispanic Black* | 0.61(0.31, 1.20)P=0.15 | 0.39(0.22, 0.70)P=0.003 | 0.24(0.13, 0.44)P<0.001 |  |
| *Other Race - Including Multi-Racial* | 0.48(0.18, 1.29)P=0.13 | 0.99(0.31, 3.12)P＞0.9 | 0.18(0.07, 0.43)P<0.001 |  |
| *Mexican American* | 0.45(0.28, 0.72)P<0.001 | 0.38(0.23, 0.64)P<0.001 | 0.15(0.08, 0.25)P<0.001 |  |
| *Other Hispanic* | 1.02(0.50, 2.12)P=0.9 | 0.41(0.19, 0.90)P= 0.028 | 0.36(0.13, 0.97)P=0.044 |  |
| **BMI (Kg/m^2^)** |  |  |  | P=0.411 |
| *Normal(<25)* | 0.51(0.33, 0.79)P=0.003 | 0.3(0.19, 0.48)P<0.001 | 0.16(0.09, 0.27)P<0.001 |  |
| *Obese(≥30)* | 0.69(0.39, 1.22) P=0.2 | 0.69(0.37, 1.29)P=0. 2 | 0.23(0.12, 0.45)P<0.001 |  |
| *Overweight(≥25,<30)* | 0.35(0.15, 0.82)P=0.016 | 0.26(0.11, 0.63)P=0.003 | 0.2(0.08, 0.52P=0.001 |  |
| **PIR** |  |  |  | P=0.125 |
| *High(>3.49)* | 0.58(0.32, 1.05)P=0.073 | 0.43(0.21, 0.87)P=0.02 | 0.13(0.06, 0.28)P<0.001 |  |
| *Low(≤1.39)* | 0.71(0.42, 1.18) P=0.2 | 0.49(0.28, 0.85)P=0.012 | 0.39(0.21, 0.76)P=0.006 |  |
| *Medium(>1.39,<=3.49)* | 0.42(0.25, 0.68)P<0.001 | 0.38(0.20, 0.74)P=0.005 | 0.21(0.11, 0.40)P<0.001 |  |
| **Sedentary. status (Minutes /day)** |  |  |  | P=0.956 |
| *Mild(<480)* | 0.59(0.42, 0.84)P=0.004 | 0.45(0.29, 0.70)P<0.001 | 0.23(0.15, 0.36)P<0.001 |  |
| *Severe(≥480)* | 0.52(0.32, 0.86)P=0.012 | 0.35(0.19, 0.65)P=0.001 | 0.18(0.10, 0.33)P<0.001 |  |
| **Drinking. status** |  |  |  | P=0.175 |
| *Current drinker* | 0.45(0.28, 0.72)P=0.001 | 0.38(0.20, 0.73)P=0.004 | 0.17(0.10, 0.31)P<0.001 |  |
| *Former drinker* | 0.49(0.30, 0.81)P=0.006 | 0.61(0.33, 1.12)P=0.022 | 0.17(0.10, 0.29)P<0.001 |  |
| *Non-drinker* | 0.77(0.47, 1.27)P=0.9 | 0.34(0.17, 0.68)P=0.003 | 0.33(0.17, 0.64)P=0.002 |  |
| **Smoking. status** |  |  |  | P=0.985 |
| *Current Smoker* | 0.71(0.22, 2.33)P=0.6 | 0.41(0.10, 1.79)P=0.2 | 0.24(0.08, 0.73)P=0.014 |  |
| *Former Smoker* | 0.46(0.20, 1.07)P=0.07 | 0.4(0.15, 1.02)P=0.055 | 0.11(0.05, 0.24)P<0.001 |  |
| *Non-Smoker* | 0.59(0.41, 0.87)P=0.007 | 0.43(0.29, 0.64)P<0.001 | 0.24(0.15, 0.37)P<0.001 |  |
| **Hypertension** |  |  |  | P=0.490 |
| *Yes* | 0.76(0.44, 1.32)P=0.3 | 0.45(0.25, 0.80)P=0.008 | 0.3(0.16, 0.59)P<0.001 |  |
| *No* | 0.5(0.33, 0.76)P=0.001 | 0.43(0.25, 0.74)P=0.003 | 0.19(0.12, 0.29)P<0.001 |  |
| **Hyperlipidemia** |  |  |  | P=0.753 |
| *Yes* | 0.99(0.29, 3.42)P>0.9 | 0.64(0.23, 1.77)P=0.4 | 0.5(0.14, 1.71)P=0.3 |  |
| *No* | 0.54(0.39, 0.74)P<0.001 | 0.45(0.17, 1.16)P=0.1 | 0.27(0.12, 0.63)P=0.003 |  |
| **CVD** |  |  |  | P=0.532 |
| *Yes* | 0.58(0.39, 0.85)P=0.003 | 0.43(0.28, 0.67)P<0.001 | 0.21(0.13, 0.33)P<0.001 |  |
| *No* | 0.45(0.22, 0.95)P=0.037 | 0.52(0.21, 1.31)P=0.2 | 0.32(0.12, 0.84)P=0.022 |  |
| **LDL-C(mmol/L)** |  |  |  | 0.169 |
| *Normal (＜2.6)* | 0.83(0.55, 1.26)P=0.4 | 1.15(0.70, 1.88)P=0.6 | 0.57(0.33, 0.96)P=0.036 |  |
| *Moderately elevated (2.6, 3.4]* | 0.93(0.53, 1.65) | 0.95(0.49, 1.82) | 0.46(0.23, 0.90)P=0.025 |  |
| *High (＞3.4)* | 0.63(0.38, 1.06) | 0.38(0.21, 0.68)P=0.002 | 0.2(0.10, 0.41) P<0.001 |  |
| **TG(mmol/L)** |  |  |  | 0.206 |
| *Normal (＜1.7)* | 0.76(0.50, 1.17)P=0.2 | 0.66(0.40, 1.09)P=0.11 | 0.41(0.26, 0.65)P<0.001 |  |
| *Moderately elevated (1.7, 2.3]* | 1.03(0.64, 1.67)P=0.9 | 0.46(0.23, 0.92)P=0.029 | 0.59(0.14, 2.54)P=0.5 |  |
| *High (＞2.3)* | 0.29(0.14, 0.61)P=0.002 | 0.61(0.19, 1.95)P=0.4 | 0.26(0.05, 1.44)P=0.12 |  |
| **TC(mmol/L)** |  |  |  | 0.352 |
| *Normal (＜5.2)* | 0.84(0.63, 1.12)P=0.2 | 0.84(0.40, 1.76)P=0.6 | 0.4(0.16, 1.00)P=0.05 |  |
| *Moderately elevated (5.2, 6.2]* | 1.34(0.87, 2.07)P=0.2 | 0.48(0.21, 1.07)P=0.072 | 0.19(0.07, 0.51)P<0.001 |  |
| *High (＞6.2)* | 0.72(0.47, 1.10)P=0.12 | 0.24(0.12, 0.49)P<0.001 | 0.11(0.04, 0.33)P<0.001 |  |

**Multiple logistic regression model:** Model 1: Adjusted for Age; Gender; Sex; Race; Model 2: Adjusted for Age; Gender; Sex; Race; Education; Marital; PIR; BMI; Sedentary behavior; Cotinine; Alcohol; Smoke; LDL-C; TC; TG; Model 3: Adjusted for Age; Gender; Sex; Race; Education; Marital; PIR; BMI; Sedentary behavior; Cotinine; Alcohol; Smoke; Hypertension; Hyperlipidemia; CVD; LDL-C; TC; TG.

**Subgroup Analysis Adjustment Factors**: Age; Gender; Sex; Race; Education; Marital status; PIR; Body Mass Index; Sedentary behavior; Simvastatin; Alcohol; Smoking; Hypertension; Hyperlipidemia; CVD; LDL-C; TC; TG, excluding sub-group variables, and the reference object in the sub-group is Q1 of HDL-C.

T2DM: type 2 diabetes mellitus; BMI: Body mass index; PIR: poverty income ratio; CVD: cardiovascular diseases; HDL-C: high-density lipoprotein cholesterol; LDL-C: Low-density lipoprotein; TG: Triglycerides; TC: Total cholesterol.

*P < 0.05; **P < 0.01; ***P < 0.001.

**Table S4. Multivariable adjusted logistic regression analysis with T2DM risk weighting for different levels of LDL-C, TG, and TC**

| **Regression model** | **Crude Model**  **OR (95% CI)** | **Model 1**  **OR (95% CI)** | **Model 2**  **OR (95% CI)** | **Model3**  **OR (95% CI)** |
| --- | --- | --- | --- | --- |
| **LDL-C(mmol/L)** |  |  |  |  |
| *Normal (＜2.6)* | Reference | Reference | Reference | Reference |
| *Moderately elevated (2.6, 3.4]* | 0.52(0.42, 0.64)*** | 0.49(0.38, 0.63)*** | 0.72(0.52, 1.01) | 0.74(0.54, 1.01) |
| *High (＞3.4)* | 0.52(0.40, 0.68) *** | 0.44(0.32, 0.60)*** | 0.98(0.55, 1.72) | 0.88(0.50, 1.54) |
| **TG(mmol/L)** |  |  |  |  |
| *Normal (＜1.7)* | Reference | Reference | Reference | Reference |
| *Moderately elevated (1.7, 2.3]* | 2.92(2.24, 3.80)*** | 2.88(2.22, 3.74)*** | 1.58(0.98, 2.55) | 1.56(0.95, 2.58) |
| *High (＞2.3)* | 3.42(2.61, 4.48)*** | 3.53(2.62, 4.76)*** | 1.12(0.48, 2.65) | 1.16(0.47, 2.90) |
| **TC(mmol/L)** |  |  |  |  |
| *Normal (＜5.2)* | Reference | Reference | Reference | Reference |
| *Moderately elevated (5.2, 6.2]* | 0.57(0.43, 0.76)*** | 0.45(0.32, 0.63) *** | 0.79(0.52, 1.21) | 0.65(0.42, 1.00) |
| *High (＞6.2)* | 0.74(0.55, 0.99)* | 0.56(0.40, 0.79) *** | 1.6(0.84, 3.04) | 1.3(0.68, 2.52) |

**Multiple logistic regression model:** Model 1: Adjusted for Age; Gender; Sex; Race; Model 2: Adjusted for Age; Gender; Sex; Race; Education; Marital; PIR; BMI; Sedentary behavior; Cotinine; Alcohol; Smoke; LDL-C; TC; TG; Model 3: Adjusted for Age; Gender; Sex; Race; Education; Marital; PIR; BMI; Sedentary behavior; Cotinine; Alcohol; Smoke; Hypertension; Hyperlipidemia; CVD; LDL-C; TC; TG; HDL-C.

T2DM: type 2 diabetes mellitus; BMI: Body mass index; PIR: poverty income ratio; CVD: cardiovascular diseases; HDL-C: high-density lipoprotein cholesterol; LDL-C: Low-density lipoprotein; TG: Triglycerides; TC: Total cholesterol.

*P < 0.05; **P < 0.01; ***P < 0.001.

**Table S5. For two-sample Mendelian randomization analysis, instrumental variables of high-density lipoprotein cholesterol.**

| SNP | Chr | Position | EA/NEA | Se | Beta | Eaf | Pval | F-statistics |
| --- | --- | --- | --- | --- | --- | --- | --- | --- |
| rs4983559 | 14 | 105277209 | G/A | 0.0036 | 0.0197 | 0.3773 | 9.57E-09 | 29.94 |
| rs12801636 | 11 | 65391317 | A/G | 0.0042 | 0.0235 | 0.2243 | 3.15E-08 | 31.31 |
| rs2602836 | 4 | 100014805 | A/G | 0.0034 | 0.0192 | 0.4274 | 4.96E-08 | 31.89 |
| rs2606736 | 3 | 11400249 | C/T | 0.0043 | 0.0246 | 0.3945 | 4.80E-08 | 32.73 |
| rs13099479 | 3 | 52677478 | A/G | 0.0062 | 0.036 | 0.08971 | 1.82E-08 | 33.71 |
| rs10761771 | 10 | 65230164 | C/T | 0.0034 | 0.0198 | 0.467 | 4.12E-09 | 33.91 |
| rs1866956 | 8 | 19748921 | T/C | 0.0037 | 0.0217 | 0.6755 | 7.96E-10 | 34.40 |
| rs10019888 | 4 | 26062990 | A/G | 0.0046 | 0.027 | 0.8364 | 4.90E-08 | 34.45 |
| rs1936800 | 6 | 127436064 | C/T | 0.0034 | 0.02 | 0.5277 | 3.05E-10 | 34.60 |
| rs13076253 | 3 | 131751775 | A/C | 0.0048 | 0.0283 | 0.8522 | 4.96E-09 | 34.76 |
| rs16965220 | 16 | 57065121 | A/C | 0.0037 | 0.0219 | 0.2982 | 7.91E-09 | 35.03 |
| rs731839 | 19 | 33899065 | A/G | 0.0037 | 0.022 | 0.6583 | 3.44E-09 | 35.35 |
| rs499974 | 11 | 75455021 | C/A | 0.0044 | 0.0263 | 0.8245 | 1.12E-08 | 35.73 |
| rs424346 | 15 | 59010962 | T/C | 0.0113 | 0.0679 | 0.04881 | 4.84E-08 | 36.11 |
| rs4917014 | 7 | 50305863 | G/T | 0.0036 | 0.0222 | 0.3404 | 1.03E-08 | 38.03 |
| rs4650994 | 1 | 178515312 | G/A | 0.0034 | 0.021 | 0.5172 | 6.70E-09 | 38.15 |
| rs11045163 | 12 | 20463526 | G/A | 0.0035 | 0.0217 | 0.4063 | 3.20E-09 | 38.44 |
| rs6567160 | 18 | 57829135 | T/C | 0.0041 | 0.0257 | 0.7691 | 2.92E-09 | 39.29 |
| rs11065987 | 12 | 112072424 | A/G | 0.0035 | 0.0222 | 0.5778 | 1.23E-09 | 40.23 |
| rs17173637 | 7 | 150529449 | T/C | 0.0057 | 0.0363 | 0.90237 | 1.90E-08 | 40.56 |
| rs2066714 | 9 | 107586753 | C/T | 0.0071 | 0.0453 | 0.1201 | 7.26E-10 | 40.71 |
| rs10087900 | 8 | 144303418 | G/A | 0.0036 | 0.0231 | 0.5607 | 2.17E-09 | 41.17 |
| rs12412743 | 10 | 114045333 | C/T | 0.0045 | 0.0291 | 0.847 | 1.31E-09 | 41.82 |
| rs6031587 | 20 | 43038249 | C/T | 0.0074 | 0.0488 | 0.9314 | 1.92E-09 | 43.49 |
| rs970548 | 10 | 46013277 | C/A | 0.0039 | 0.0258 | 0.277 | 1.71E-10 | 43.76 |
| rs1980493 | 6 | 32363215 | T/C | 0.0048 | 0.0318 | 0.8773 | 3.76E-10 | 43.89 |
| rs3861397 | 6 | 139828916 | A/G | 0.0036 | 0.024 | 0.6583 | 8.40E-11 | 44.44 |
| rs998584 | 6 | 43757896 | C/A | 0.0038 | 0.026 | 0.4855 | 2.27E-11 | 46.81 |
| rs4379922 | 12 | 125351116 | C/T | 0.0036 | 0.0247 | 0.3496 | 9.56E-12 | 47.07 |
| rs1047891 | 2 | 211540507 | C/A | 0.0039 | 0.0269 | 0.6979 | 8.73E-10 | 47.57 |
| rs12133576 | 1 | 93816400 | A/G | 0.0035 | 0.0243 | 0.3549 | 6.15E-11 | 48.20 |
| rs2013208 | 3 | 50129399 | T/C | 0.0036 | 0.0254 | 0.5053 | 8.92E-12 | 49.78 |
| rs702485 | 7 | 6449272 | G/A | 0.0034 | 0.0243 | 0.4499 | 6.45E-12 | 51.08 |
| rs205262 | 6 | 34563164 | A/G | 0.0039 | 0.0283 | 0.7335 | 3.88E-13 | 52.65 |
| rs492571 | 15 | 44211273 | T/C | 0.009 | 0.0663 | 0.95778 | 1.27E-12 | 54.27 |
| rs3741414 | 12 | 57844049 | T/C | 0.004 | 0.0296 | 0.1913 | 6.10E-14 | 54.76 |
| rs4969178 | 17 | 76388202 | G/A | 0.0035 | 0.0263 | 0.6266 | 1.53E-12 | 56.46 |
| rs687339 | 3 | 135932359 | C/T | 0.0042 | 0.0316 | 0.2335 | 7.11E-13 | 56.61 |
| rs9457931 | 6 | 160929904 | A/G | 0.0073 | 0.0552 | 0.9314 | 7.30E-13 | 57.18 |
| rs17145738 | 7 | 72982874 | T/C | 0.0053 | 0.0408 | 0.1174 | 4.95E-13 | 59.26 |
| rs2642438 | 1 | 220970028 | G/A | 0.0039 | 0.0303 | 0.7454 | 7.78E-14 | 60.36 |
| rs4148005 | 17 | 66882466 | T/G | 0.0036 | 0.0283 | 0.7005 | 5.74E-14 | 61.80 |
| rs2454722 | 12 | 123171218 | G/A | 0.0044 | 0.0351 | 0.1451 | 3.31E-14 | 63.64 |
| rs12748152 | 1 | 27138393 | C/T | 0.0062 | 0.0506 | 0.92876 | 9.74E-16 | 66.61 |
| rs2293889 | 8 | 116599199 | G/T | 0.0035 | 0.0312 | 0.5871 | 4.27E-17 | 79.46 |
| rs2250802 | 10 | 113921354 | G/A | 0.0038 | 0.034 | 0.3193 | 2.02E-17 | 80.05 |
| rs181360 | 22 | 21928916 | T/G | 0.0042 | 0.0376 | 0.8008 | 9.24E-18 | 80.14 |
| rs737337 | 19 | 11347493 | T/C | 0.0061 | 0.0565 | 0.9314 | 4.56E-17 | 85.79 |
| rs1877031 | 17 | 37814080 | A/G | 0.0036 | 0.0336 | 0.6755 | 1.20E-19 | 87.11 |
| rs2278236 | 19 | 8431581 | A/G | 0.0035 | 0.0331 | 0.5435 | 3.18E-18 | 89.44 |
| rs2241210 | 12 | 109950144 | G/A | 0.0035 | 0.0332 | 0.5528 | 2.49E-20 | 89.98 |
| rs7306660 | 12 | 125327384 | G/A | 0.0036 | 0.0345 | 0.6306 | 3.34E-19 | 91.84 |
| rs11789603 | 9 | 107647019 | T/C | 0.006 | 0.06 | 0.08971 | 3.69E-21 | 100.00 |
| rs686030 | 9 | 15304782 | A/C | 0.0049 | 0.055 | 0.8588 | 4.29E-27 | 125.99 |
| rs10808546 | 8 | 126495818 | T/C | 0.0034 | 0.0409 | 0.4459 | 4.11E-30 | 144.71 |
| rs838876 | 12 | 125259888 | A/G | 0.0039 | 0.0493 | 0.3259 | 7.32E-33 | 159.79 |
| rs4846914 | 1 | 230295691 | A/G | 0.0034 | 0.0479 | 0.5844 | 3.51E-41 | 198.48 |
| rs4240624 | 8 | 9184231 | A/G | 0.0058 | 0.0818 | 0.9248 | 1.32E-45 | 198.91 |
| rs16942887 | 16 | 67928042 | A/G | 0.0051 | 0.0831 | 0.1332 | 8.28E-54 | 265.50 |
| rs633695 | 15 | 58725839 | G/A | 0.0054 | 0.0885 | 0.285 | 7.82E-58 | 268.59 |
| rs676210 | 2 | 21231524 | A/G | 0.004 | 0.066 | 0.2309 | 2.34E-54 | 272.25 |
| rs2241770 | 16 | 56866196 | T/C | 0.0057 | 0.0989 | 0.8971 | 6.78E-60 | 301.05 |
| rs4939883 | 18 | 47167214 | C/T | 0.0045 | 0.0799 | 0.8193 | 1.80E-66 | 315.26 |
| rs13702 | 8 | 19824492 | C/T | 0.0038 | 0.1058 | 0.3127 | 1.28E-160 | 775.17 |
| rs10468017 | 15 | 58678512 | T/C | 0.0038 | 0.1179 | 0.2757 | 1.21E-188 | 962.62 |

Chr, chromosome; EA/NEA: effect allele/other allele; Eaf: effect allele frequency

**Table S6. Multivariate Mendelian randomization of HDL-C and T2DM after adjustment for other lipid traits**

| **Exposure** | **Outcome** | **SNP** | **OR (95% CI)** | **P** |
| --- | --- | --- | --- | --- |
| HDL-C | Type 2 diabetes  (T2DM) | 77 | 0.41(0.14, 0.68) | 8.02× 10^-3^ |
| LDL-C |  | 69 | 0.61(0.14, 1.30) | 0.070 |
| TG |  | 41 | 2.19(0.84, 3.68) | 0.101 |
| TC |  | 76 | 0.63(0.27, 1.08) | 0.066 |

Low-density lipoprotein (LDL) (n = 173,820), triglycerides (TG) (n = 177,861), and total cholesterol (TC) (n = 187,365), along with high-density lipoprotein (HDL-C), were sourced from a global lipid genetics consortium (GLGC) for a comprehensive whole-genome association study (GWAS). To create genetic instrumental variables (IVs) for circulating lipids, we identified variants with genome-wide significance for the three lipid traits (P < 5×10^−8^), with a linkage disequilibrium threshold (LD r^2^≤0.001) and a minimum physical distance of 10 Mb in the GWAS summary statistics.

**Table S7. Causality and sensitivity analysis of T2DM and HDL-C in reverse two-sample Mendelian randomization analysis**

| **Exposure** | **Outcome** | **SNP** | **Methods** | **OR (95% CI)** | **P** |
| --- | --- | --- | --- | --- | --- |
| Type 2 diabetes  (T2DM) | High density lipoprotein cholesterol  (HDL-C) | 6 | MR Egger | 0.98(0.76,1.26) | 0.889 |
|  |  |  | Weighted median | 1.01(0.97,1.04) | 0.778 |
|  |  |  | Inverse variance weighted | 1.00(0.97,1.03) | 0.887 |
|  |  |  | Simple mode | 1.01(0.96,1.06) | 0.698 |
|  |  |  | Weighted mode | 1.01(0.97,1.05) | 0.723 |
|  |  |  | **Cochran Q- P.value** | **MR-Egger Intercept-P.value** | **MR-PRESSO- P.value** |
|  |  |  | 0.851 | 0.901 | 0.873 |
